# Use of varenicline for smoking cessation treatment in UK primary care: an association rule mining analysis

**DOI:** 10.1186/1471-2458-14-1024

**Published:** 2014-10-02

**Authors:** Yue Huang, Sarah Lewis, John Britton

**Affiliations:** UK Centre for Tobacco and Alcohol Studies, Division of Epidemiology and Public Health, University of Nottingham, Clinical Sciences Building, City Hospital, Nottingham, NG5 1PB UK

**Keywords:** Varenicline, Primary care data, Smoking cessation, Associate rule mining

## Abstract

**Background:**

Varenicline is probably the most effective smoking cessation pharmacotherapy, but is less widely used than nicotine replacement therapy. We therefore set out to identify the characteristics of numerically important groups of patients who typically do, or do not, receive varenicline in the UK.

**Methods:**

We used association rule mining to analyse data on prescribing of smoking cessation pharmacotherapy in relation to age, sex, comorbidity and other variables from 477,620 people aged 16 years and over, registered as patients throughout 2011 with one of 559 UK general practices in The Health Improvement Network (THIN) database, and recorded to be current smokers.

**Results:**

46,685 participants (9.8% of all current smokers) were prescribed any smoking cessation treatment during 2011, and 19,316 of these (4% of current smokers, 41% of those who received any therapy) were prescribed varenicline. Prescription of varenicline was most common among heavy smokers aged 31–60, and in those with a diagnosis of COPD. Varenicline was rarely used among smokers who were otherwise in good health, or were aged over 60, were lighter smokers, or had psychotic disorders or dementia.

**Conclusions:**

Varenicline is being underused in healthy smokers, or in older smokers, and in those with psychotic disorders or dementia. Since varenicline is probably the most effective available single cessation therapy, this study identifies under-treatment of substantial public health significance.

## Background

Smoking is the largest avoidable cause of death in the UK. About half of all life-long smokers will die prematurely, losing on average about 10 years of life [1, 2]. There were a total of 442,759 deaths of adults aged 35 and over in England of which 79,100 (18%) were estimated to be attributable to smoking in 2011 [3].

Smoking cessation treatments are one of the most cost effective of all health care treatments. Of available pharmacotherapies, NRT and varenicline are most widely used. Varenicline is a competitive nicotine receptor antagonist with partial agonist activity that has been available as a smoking cessation treatment since 2006. Its targeted mechanism of action, better efficacy and tolerability makes varenicline a useful therapeutic option for smoking cessation [4]. Varenicline therapy increases the likelihood of sustained cessation relative to placebo by a ratio of around 2.3, and may be more effective than conventional Nicotine Replacement Therapy (NRT) [5]. However, whilst use of varenicline has been approved by NICE since 2008 [6], it has not been as widely used as NRT [7]. Available clinical-trial data [8] has supported varenicline as a first-line treatment option for smoking cessation in healthy adult smokers, but to our knowledge, the use of it in the general population, particularly those with comorbid conditions has not been reported.

We have previously used data mining methods in a large nationally representative primary care database to demonstrate that there are identifiable groups of patients who are systematically failing to be offered any treatment for smoking cessation in UK primary care, notably older smokers and those with dementia or hypertension [9]. We have now used the same methods to investigate the characteristics of smokers who do and do not receive prescriptions for varenicline, using data from a nationally representative primary care database for 2011.

## Methods

### Study population

We used data from The Health Improvement Network (THIN), a large electronic anonymised primary care database, with records from 559 general practices across the UK. We identified for analysis all individuals aged 16 years or over who had been registered with a THIN practice throughout 2011, and whose record indicated that they were a current smoker, either having a Read code indicative of current smoking recorded during 2011, or in those with no smoking status recorded in 2011, since 1^st^ Oct 2009. Since the Quality and Outcomes Framework (QOF) requires general practitioners (GPs) to record the smoking status of their patients at least every 27 months, all individuals should have had a smoking status recorded in this timeframe [10].

For each of these patients, we identified whether they had received a prescription for varenicline during 2011, using drug codes from BNF. We also extracted data on age (categorised as 16 to 30, 31 to 45, 46 to 60 and 61 or over), gender, whether patients were coded as heavy smokers (>20 cigarettes/day), had alcohol weekly consumption over recommended levels (>14 units/week for women and 21 units/week for men) and obesity (body mass index >30). We extracted data on medical conditions based on the clinical disease indicators in the Quality and Outcomes Framework (QOF), including asthma, atrial fibrillation, cancer, coronary heart disease (CHD), chronic kidney disease (CKD), chronic obstructive pulmonary disease (COPD), dementia, anxiety or depression, diabetes, epilepsy, heart failure, hypertension, learning disability, psychotic disorders, stroke or transient ischaemic attack (TIA) and thyroid disorders. Any diagnosis made before the end of 2011 was included.

### Ethics statement

Ethical approval for the use of THIN data for this study was obtained from the THIN Scientific Review Committee (reference number 10–022A).

### Analysis

We used association rule mining (ARM) to discover the combined characteristics of groups of patients who did, or did not receive a varenicline prescription. ARM is one of the most well-established techniques in data mining, and has been widely applied in many areas. ARM identifies rules of the form ***A*** = > ***B***, where ***A*** is the subset of exposures, and ***B*** is the subset of outcomes. ***A*** and ***B*** are mutually exclusive. We used the ARM method to identify rules for sets of characteristics (***A***) of patients who did, and did not, receive a prescription for varenicline during 2011 (***B***), carrying out two separate analyses for these two different outcomes (receiving varenicline/NOT receiving varenicline). ARM rules are produced to meet minimum constraints on level of ‘support’ for the rule, that is, the proportion or number of the study population, in this case current smokers, with the characteristics in set ***A***. Since we were interested in identifying numerically important exposure groups while also ensuring that each of the patient groups represented by the disease indicators had potential to be included in our rules, we set a minimum exposure group size of 500 to ensure that exposure groups were numerically important. The rules were listed in order of ‘confidence’, that is the probability of ***B*** given ***A***, or the probability of receiving, or not receiving, a prescription given that the patient has each of the characteristics in the set ***A***. A more detailed description of our ARM approach was provided in our previous study which investigated the characteristics of smokers who received any smoking cessation prescription in primary care [9]. We used the open-source data mining software WEKA [11] to undertake the ARM analyses.

## Results

A total of 477,620 smokers were identified and included in the analysis. Of these, 46,685 (9.8%) received a prescription for one or more smoking cessation medication during 2011, and 19,316, 4.0% of all smokers, were prescribed varenicline (Table 1).

**Table 1:** **The description of the study population**

	Total		Receiving varenicline	
	n	%	n	%
**Total**	477620		19316	4.04
**Age groups, years**				
**16-30**	126382	26.5	3593	2.84
**31-45**	139778	29.3	7004	5.01
**46-60**	125127	26.2	6118	4.89
**>60**	86333	18.1	2601	3.01
**Sex**				
**Male**	237709	50.0	7861	3.57
**Female**	239911	50.0	8730	4.05
**Lifestyle**				
**Alcohol**	15132	3.2	457	3.02
**Heavy smoker**	56619	11.9	4564	8.06
**Obesity**	45805	9.6	1901	4.15
**Medical condition**				
**Asthma**	71759	15.0	3494	4.87
**Atrial fibrillation**	4629	1.0	105	2.27
**Cancer**	10862	2.3	457	4.21
**CHD**	17271	3.6	706	4.09
**CKD**	12313	2.6	307	2.49
**COPD**	22227	4.7	1511	6.80
**Dementia**	4198	0.9	79	1.88
**Depression/anxiety**	144520	30.3	6202	4.29
**Diabetes**	22817	4.8	931	4.08
**Epilepsy**	5433	1.1	108	1.99
**Heart failure**	3452	0.7	78	2.26
**Hypertension**	59505	12.5	2054	3.45
**Learning disability**	1239	0.3	15	1.21
**Psychotic disorders**	7544	1.6	81	1.07
**Stroke or TIA**	10979	2.3	430	3.92
**Thyroid**	19394	4.1	818	4.22

The patients identified by the top 10 rules (Table 2) for receiving a varenicline prescription were all heavy smokers aged between 31 and 60 who were not heavy drinkers, were not obese and did not have hypertension. Some also had COPD (rules 1.9 and 1.10). They did not have mental health problems including anxiety or depression, psychotic disorders and dementia; and did not have epilepsy. In these groups of patients, confidence was 13.3% or more, so the proportions receiving a varenicline prescription was much higher than the 4% in the general population of smokers.

**Table 2:** **Top 10 generated rules for smokers prescribed varenicline & NOT prescribed varenicline (exposure group size > =500)**

Outcome = Prescribed varenicline			Outcome = NOT prescribed varenicline		
(Analysis 1)			(Analysis 2)		
Exposure1		Confidence (%)	Exposure2		Confidence (%)
1.1	alcohol = NO, heavy smoker = YES, obesity = NO, dementia = NO, depression/anxiety = NO, epilepsy = NO, hypertension = NO, psychotic disorders = NO, age = 31-45, gender = female	14.9	2.1	psychotic disorders = YES, age > 60, sex = female	99.7
1.2	alcohol = NO, heavy smoker = YES, obesity = NO, depression/anxiety = NO, epilepsy = NO, hypertension = NO, psychotic disorders = NO, age = 31-45, gender = female	14.8	2.2	COPD = NO, psychotic disorders = YES, age > 60, gender = female	99.6
1.3	alcohol = NO, heavy smoker = YES, obesity = NO, dementia = NO, depression/anxiety = NO, epilepsy = NO, hypertension = NO, psychotic disorders = NO, age = 31-45	14.0	2.3	alcohol = YES, psychotic disorders = YES	99.6
1.4	alcohol = NO, heavy smoker = YES, obesity = NO, depression/anxiety = NO, epilepsy = NO, hypertension = NO, psychotic disorders = NO, age = 31-45	14.0	2.4	psychotic disorders = YES, age > 60	99.5
1.5	alcohol = NO, heavy smoker = YES, obesity = NO, dementia = NO, depression/anxiety = NO, hypertension = NO, psychotic disorders = NO, age = 31-45	13.9	2.5	heavy smoker = NO, COPD = NO, dementia = YES, age > 60	99.5
1.6	alcohol = NO, heavy smoker = YES, obesity = NO, depression/anxiety = NO, hypertension = NO, psychotic disorders = NO, age = 31-45	13.8	2.6	heavy smoker = NO, psychotic disorders = YES, age > 60	99.4
1.7	alcohol = NO, heavy smoker = YES, obesity = NO, dementia = NO, depression/anxiety = NO, epilepsy = NO, hypertension = NO, psychotic disorders = NO, age = 31-45, gender = male	13.5	2.7	COPD = NO, psychotic disorders = YES, age > 60	99.4
1.8	alcohol = NO, heavy smoker = YES, obesity = NO, depression/anxiety = NO, epilepsy = NO, hypertension = NO, psychotic disorders = NO, age = 31-45, gender = male	13.4	2.8	heavy smoker = NO, dementia = YES, depression/anxiety = NO, age > 60	99.4
1.9	alcohol = NO, heavy smoker = YES, obesity = NO, asthma = YES, CKD = 0, COPD = YES, dementia = NO, epilepsy = NO, hypertension = NO, learning disability = NO, psychotic disorders = NO, age = 46-60	13.4	2.9	heavy smoker = NO, COPD = NO, psychotic disorders = YES, age > 60	99.3
1.10	alcohol = NO, heavy smoker = YES, obesity = NO, COPD = YES, dementia = NO, depression/anxiety = NO, epilepsy = NO, hypertension = NO, psychotic disorders = NO, age = 46-60	13.3	2.10	heavy smoker = NO, psychotic disorders = YES, age = 31-45, gender = male	99.3

When we applied the same approach to identify those who did not receive varenicline, we initially generated a large number of rules of the form ‘one or more disease/lifestyle indicator = NO’, and their confidence levels were all very high (>97%, most of them 100%), indicating that non-heavy smokers in good health are very unlikely to receive varenicline. To explore prescribing in those with other co-morbidity, therefore we reset the constraints for rule generation such that our disease indicators were restricted to those with at least one comorbidity. The top 10 rules for smokers who did not receive a varenicline prescription are also shown in Table 2 (analysis 2). In these smokers, 8 out of 10 rules define groups of patients with psychotic disorders, and the other 2 defined groups with dementia. Most of the groups defined by these rules were patients aged over 60 who were not heavy smokers and/or did not have COPD. These were groups in which confidence was 99.3% or more, so that 0.7% or less were receiving a prescription.

## Discussion

To our knowledge, this is the first study to investigate the prescribing of varenicline for smoking cessation treatment in UK general practice. The advantage of using the ARM method is that it allows us to approach data with no prior hypothesis or null hypothesis, and to identify the combinations of characteristics that define those who are especially likely or unlikely to get a prescription. Our study demonstrates that only about one in every 25 current smokers ascertained by general practitioners were prescribed varenicline during the one year study period, while ARM analysis demonstrates that varenicline was prescribed preferentially to heavy smokers who were otherwise healthy, or who had COPD. The most notable groups who were not prescribed varenicline were those who were older, and those with mental health conditions, particularly psychotic disorders or dementia. Varenicline was also less likely to be prescribed in lighter smokers, and in people with epilepsy, atrial fibrillation or hypertension.

People with severe mental health problems are more likely to smoke and to smoke more heavily, are less likely to quit smoking, and experience a very high risk of death or morbidity due to smoking [12]. However this group is generally under-treated for smoking cessation in primary care given their consultation rate [2]. The latest study shows patients with mental health problems can benefit from stopping smoking [13], and health professionals have been encouraged to provide their patients with the same level of smoking cessation support that is given to the rest of the population [14–16]. Use of varenicline has been inhibited by concerns over adverse events, particularly depression and suicidal ideation [5, 17, 18], with the result that this treatment may be withheld from smokers perceived to be at particular risk of these effects, such as those with existing mental health problems. However, recent evidence suggests that these concerns are unfounded [19], and both the BNF and National Institute for Health and Care Excellence (NICE) guidance indicate that the risk should be managed by clinical monitoring, not by non-use [6, 16, 18]. Since continued smoking carries a more substantial health risk for the great majority of these individuals, this practice may be counterproductive to individual and public health. In recent trials, varenicline has been shown to increase smoking cessation in smokers with severe mental health problems without worsening their mental health condition, and help to reduce the high prevalence of tobacco dependence and the heavy burden of smoking-related morbidity and mortality within this population [20, 21].

Smoking is also a risk factor for dementia [22] and smoking cessation is recommended in secondary prevention of the condition [15]. Our findings suggest that people with dementia are very unlikely to receive varenicline, one of the most effective cessation therapies, from their GP. Any risks of adverse effects with smoking cessation therapies must be balanced against the risk of continued smoking, which for most users is the likely long-term untreated outcome.

## Conclusion

In general, the message from ARM indicates that healthy individuals are more likely to receive varenicline; on the contrary, those smokers with more co-morbidity are less likely to get varenicline. The balance of risks between long-term sustained smoking and short term adverse effects of varenicline therapy suggests however that more widespread use of varenicline could deliver substantial individual and population health benefits. There is a case for wider use of varenicline in the smoking population.

## Abbreviations

ARM: Associate Rule Mining
CHD: Coronary Heart Disease
CKD: Chronic Kidney Disease
COPD: Chronic Obstructive Pulmonary Disease
NICE: National Institute for Health and Clinical Excellence
NRT: Nicotine Replacement Therapy
QOF: Quality and Outcomes Framework
THIN: The Health Improvement Network
TIA: Transient Ischaemic Attack.
